# Study of the Soil Isolates for Antimicrobial Activity

**DOI:** 10.4103/0250-474X.49132

**Published:** 2008

**Authors:** A. R. Srividya, G. S. Saritha, B. Suresh

**Affiliations:** Department of Pharmaceutical Biotechnology, J. S. S College of Pharmacy, Ooty-643 001, India

**Keywords:** Antimicrobial compounds, fermentation, International *Streptomycetes* project (ISP), *Streptomycetes* spp, melanoid formation, milk coagulation, peptonisation

## Abstract

During the process of screening for a potent antimicrobial compound, a new strain was isolated from the soil sample of Thalaikunda village in Ooty, Tamil Nadu. That organism was name as NK_2_. It was found to be antagonistic to both bacterial and fungal test organisms. Production of antibiotic was more in a newly formulated broth. Antibiotic production reached maximum at the end of the 70 h of fermentation by stirred flask culture. The antimicrobial compound was extracted in *n*-butanol, ethyl acetate and methanol. Antimicrobial compound, which was produced by the soil isolate NK_2_ did not showed cytotoxic activity on Vero cell lines.

Over five thousand antibiotics have been identified from the culture of gram-positive, gram-negative and filamentous fungi but only hundred antibiotics have been commercially used to treat human, animal and plant disease^1^. A major feature of industrial antibiotic production is directed to screening programmes for new potent antibiotic producing organism either from natural sources or from established cultures. Screening for antibiotics producing microorganism, can be detected and isolated by the use of highly selective procedure which allows detection and isolation of only those microorganism of interest from a large population is possible. Soil is the largest source of microorganisms^2^. Majority of antibiotics so far isolated were produced from *Streptomycetes*, which are common inhabitants of the soil^3^. There are 23,000 known secondary metabolite, 42% of which are produced by *Actinobacteria,* 42% by fungi (*Penicillium* spp) and 16% by other bacteria^3^ *Streptomycetes* spp. as the microorganisms become resistant after some time to a particular antibiotic, it is becoming necessary to find newer antibiotics to which the microorganism is sensitive^4^. In the present study, some bacteria and fungal strains were tested for antibiotic sensitivity. Soil isolate named as NK_2_ was found to be active on the selected microorganisms. Taxonomical studies were performed. The secondary metabolite of the soil isolate NK_2_ showed antimicrobial activity. In this paper isolation, characterization, bioprocessing and evaluation of product, obtained from the soil isolate, NK_2_ are described.

The cultures of tested microorganisms were obtained from National collection of industrial microorganisms, Pune. Vero cell lines were obtained from the Pasteur Institute of India, Coonoor. Media such as International Streptomycetes Project media (Internationally accepted Universal media for Streptomycetes), streptomycetes media (common media), nutrient agar media, Sabouraud dextrose agar and the ingredients which were used in the formulation of media were purchased from Hi-Media Laboratories, Mumbai. Various solvents used in this study were purchased from Ranbaxy Lab Ltd, SAS Nagar. Starch casein media was used for maintaining the culture NK_2_^5^.

The soil samples were collected from Thalaikunda village, Ooty, Tamil Nadu and screened for *actinomycetes,* which are capable of producing antibacterial substances. The NK_2_ isolate, showed antimicrobial activity against *Escherichia coli, Pseudomonaous aeruginosa, Staphylococcus aureus, Bacillus subtilis, Aspergillus niger, Aspergillus flavus, Candida albicans* and *Candida krusei* using the supernatant form of broth of NK_2_ over nutrient agar medium by Agar cup plate techniques^6^,^7^.

Fermentation media for NK_2_ was formulated based upon carbon utilization pattern^8^. A new fermentation medium consisting of soluble starch- 20 g, sucrose- 15 g, glucose- 5, soya bean meal- 20 g, yeast extract powder- 5 g, CaCO_3_- 3.2 g, MgSO_4_.7H_2_ O- 2.5 g, K_2_HPO_4_- 5 g, MnCl_2_- 0.2 g, NaCl- 0.01 g, FeSO_4_.4 H_2_O- 0.002 g and silicone oil as an antifoaming agent- 0.3 ml/ l. The seeded medium consisted of glucose- 10 g, soluble starch- 10 g, yeast extract powder- 5 g, beef extract- 3 g, CaCO_3_- 2 g/l. A 250 ml conical flask containing 10 ml of seed medium was inoculated with a loopful growth of the selected strain grown on slants. The flask was incubated at 28° for 48 h. Ten millilitres of NK_2_ seed culture was transferred to a 1 l conical flask containing 100 ml of the same medium and then it was incubated at 28°, the second stage seed culture was used as the inoculum to initiate the fermentation in a 5 l containing 3 l of fermentation medium. The fermentation was carried out at 28° with sufficient aeration and agitation at 200 rpm until the pH reached neutrality. Culture growth was evaluated by centrifuging the fermented broth for 10 min at 5000 revolution per minute^9^. The percentage of packed cell volume, change in pH, antibiotic production was noted.

Soil isolate, NK_2_, was studied for its morphology and its characteristics by agar block method. The morphology was observed under scanning electron microscopy (fig. 1) Carbon utilization studies were performed and based on the new fermentation medium was formulated^9^. Development of melanin pigment in Wakesman No. 42 medium was evaluated by streaming the soil isolate on the slants followed by incubation at 28° and the results were observed at the interval of 12 h for 4 days and it was recorded^10^–^13^.

**Fig. 1:**
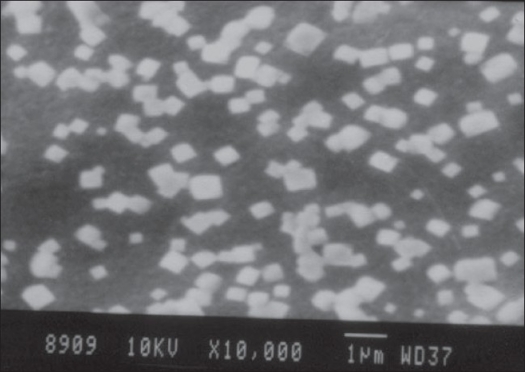
Scanning electron microscopy of NK_2_ Structure of the soil isolate NK_2_ under 10, 000 times magnification

Nitrate reducing property was evaluated by inoculating the isolate in organic nitrate broth and incubated at 28° for 5 days. From the 5^th^ day, the cultures were observed for the nitrate reduction by using the reagents such as α-naphthal solution and sulphonilic acid. Development of the pink colour indicated the nitrate reducing property of the isolate^10^–^14^. Proteolytic activity of the isolate was evaluated by inoculating them in pasteurized milk and observing for the reduction of litmus paper, formation of white band, change in pH, formation of whey like brownish translucent band and gas formation up to 48 h^10^–^13^. Presence or proteolytic enzymes was determined by growing the soil isolate over the starch agar medium and incubated at 28° for 5-7 days. The development of clear zone indicated the hydrolysis of starch and it was flooded with Lugol's iodine solution for confirmation^10^–^13^.

The soil isolate was streaked and incubated for 4 days at 28° in a medium containing ferric ammonium citrate, dibasic potassium phosphate, Na_2_S_2_O_4_, yeast extract and agar^10^–^13^. The Nutrient gelatin medium was employed to grow the soil isolate. The protein gelatin is expected to be hydrolyzed by exoenzyme, if secreted by the isolate. The solid character of the medium depends on gelatin remained in the gel state^10^–^13^. The isolate was inoculated in glucose nutrient broth along with bromothymol blue as indicator and incubated at 28° for 15 days. At every 12 h of interval, change in colour was noted^10^–^13^.

Inoculum (24 h old) was used to seed the flask at 10 % level. Fermentation was carried out for 5 d with 200 rpm at 28°. The active constituents were extracted from both filtrate and mycelia after separation by centrifugation from the fermented cultured broth. One part of the filtrate was extracted three times with equal volume of *n*-butanol and another part with ethyl acetate. The mycelia were extracted with methanol. All the three extracts were concentrated at 40° to obtain crude extracts. All the crude extracts of NK_2_ obtained from the fermented media were subjected to chromatographic analysis. Based on R_f_ values, crude antibiotic fractions were classified^14^,^15^.

Test bacteria were grown on nutrient agar and fungi were grown on Sabouraud dextrose agar medium. The extracts were dissolved in corresponding solvents and 100 μl of the samples were placed in to the corresponding cups. Zone of inhibition was measured after 24 h incubation at 37° for bacteria and after 48 h incubation at 28° for fungi. The antimicrobial activity was estimated by measuring the diameter of the inhibitory zone^16^. All extracts obtained from broth culture of NK_2_ were tested for its cytotoxic activity on Vero cell lines using the Trypan Blue exclusion techniques^9^. The samples were tested at various concentrations between 125-500 μl/ml.

In the process of screening of soil actinomycetes, the isolate NK_2_ was found to be capable of producing antibiotic against bacteria and fungi. The soil isolate NK_2_ gave slightly positive results for the nitrate reduction and starch hydrolysis and it gave intense results for the acid production test. It does not have the capacity to produce H_2_S and Melanin pigments. NK_2_ showed white band and solid formation after 24 h for milk coagulation test and at 48 h it showed white band, more whey like brownish medium, solid formation and gas formation. NK_2_-isolate showed growth in medium containing lactose, good growth in medium containing sucrose, fructose and d(+)sorbitol. it showed good growth with fermentation in the medium containing glucose and maltose and good growth with no fermentation in the medium containing d(+)mannitol.

Scanning electron microscope revealed a rectangular shape with irregular grouping of the NK_2_ isolate fig. 1. The color of the aerial mycelium was cream in ISP-2 (YEME) yeast extract malt extract agar, ISP4 (inorganic salt starch agar), ISP6 (peptone yeast extract agar) ISP7 (tyrosine agar) and it was white in ISP-3 (oats meal agar and ISP 5 (glycerol asparagine agar). NK_2_ isolate showed brown colour soluble pigments in ISP–4 medium.

The production of antibiotic was carried out in stirred flask culture. The production began after inoculations, gradually reached the maximum at 70 h and slowly decreased. At 70 h the pH was 6.8 and there was slight increase to 7. Biomass reached the maximum at 94 h and then remained at the same level till 118^th^ h. Fermentation parameters are listed in Table 1. After extraction with *n*-butanol, the fermented broth gave a cream colour powder with the percentage of yield 0.689%. Extraction with ethyl acetate yielded a brownish yellow powder with a yield of 0.0560% and methanol extract yielded a yellowish brown powder with a yield of 0.0548%. By trial and error method the optimal solvent system for TLC studies of NK_2_ was found to be butanol, acetic acid and water in the ratio of 9:0.5:0.5. The R_f_ (Retardation factor) values for the *n*-butanol, ethyl acetate and methanol was found to be 0.36, 0.08, 0.30, respectively.

**TABLE 1:** FERMENTATION PARAMETER OF SOIL ISOLATE, NK_2_

Time (h)	*Broth packed cell weight (g)	Packed cell volume (%)	pH
14	0.02445	22	6.0
22	0.02489	25	6.5
38	0.02528	29	6.5
46	0.02576	32	6.0
62	0.02593	35	6.5
70	0.02634	39	6.8
86	0.02687	43	6.8
94	0.02719	46	7
110	0.02735	48	7
118	0.02745	49	7

* Weight of broth prior to fermentation = 0.02418 g / ml.

**TABLE 2:** MICROBIAL SENSITIVITY OF THE VARIOUS EXTRACTS OF NK_2_ SOIL ISOLATES

Organisms	Zone of Inhibition (mm)		
	
	*n*-Butanol extract (100 μg/ml)	Ethyl acetate extract (100 μg/ml)	Methanol Extract (100 μg/ml)
*Escherichia coli*	33	25	24
*Pseudomonas aeruginosa*	-	-	-
*Bacillus subtilis*	32	14	14
*Staphylococcus aureus*	35	24	19
*Aspergillus niger*	-	-	22
*Aspergillus flavus*	-	-	-
*Candia albicans*	-	27	21
*Candida krusei*	-	-	-

**TABLE 3:** BIOCHEMICAL AND TAXONOMICAL RESULTS FOR NK_2_ SOIL ISOLATES

Test performed			Results obtained	
Biochemical tests				
Melanin formation			-	
Nitrate reduction			+	
H_2_S production			-	
Starch hydrolysis			+	
Gelatin liquification			-	
Acid production			++	
Milk coagulation and peptonisation				
After 24 hours			WB, S	
After 48 hours			WB, More WLB, S, G pH 7.0	
Carbon utilization tests				
Sucrose			++	
Lactose			+	
Dextro mannitol			± ±	
Dextro Sorbitol			+ +	
Glucose			+ + + +	
Maltose			+ + + +	
Fructose			+ +	
Culture characteristics				
ISP-2 media	G: Good	AM: Cream	R: Cream	Sp: Nil
ISP-3 media	G: Good	AM: White	R: Off white	Sp: Nil
ISP-4 media	G: Fair	AM: Cream	R: Cream	Sp: Brown
ISP-5 media	G: Fair	AM: White	R: White	Sp: Nil
ISP-6 media	G: Fair	AM: Cream	R: Yellow	Sp: Nil
ISP-7 media	G: Fair	AM: Cream	R: Cream	Sp: Nil

For the biochemical tests: ++ denotes very intense, + denotes slightly formed and - denotes a negative result. Milk coagulation and peptonisation test results indicated by WB, white band; G, gas formed; WLB, whey like brownish medium and S, solid formation. For the litmus paper test, purple indicates alkalinity while pink indicates acidity. In the carbon utilization tests, + denotes growth, ++ denotes good growth, +++ indicate growth with fermentation, ++++ indicates good growth with fermentation, while ±± denotes growth with no fermentation and ±±± indicates good growth with no fermentation. Culture characteristics are describes as G for growth, AM for aerial mycelium, R for reverse colour and Sp for soluble pigment.

*n*-Butanol extract from the fermented media showed the antimicrobial activity against *Escherichia coli, Staphylococcus aureus,* and *Bacillus subtilis.* Ethyl acetate extract showed the antimicrobial activity against *Escherichia coli, Staphylococcus aureus, Bacillus subtilis, Candida albicans, Candida krusei E. coli, B. subtilis, S. aureus* and *Candida albicans Ca 27*. The methanol extract showed activity against *Escherichia coli, Staphylococcus aureus, Bacillus subtilis, Aspergillus niger, Candida albicans* Ca 27. The antimicrobial activity of *n*-butanol, ethyl acetate and methanol from the stirred flask culture of NK_2_ is summarized in Table 2.

The fractions that obtained from the isolate were found to be effective against *Escherichia coli, Staphylococcus aureus, Bacillus subtilis, Candida albicans, Candida kruse, Aspergillus niger.* Based up on the antimicrobial studies and taxonomical studies (Table 3), it concluded that compound obtained from the isolate was an antibiotic belongs to the *Streptomycetes* spp. Further work can be continued for the structural elucidation of the compounds obtained by the fermentation process and it can be compared with the standard antibiotics to prove its potency.
